# Author Correction: High-harmonic generation from a flat liquid-sheet plasma mirror

**DOI:** 10.1038/s41467-023-38696-y

**Published:** 2023-05-19

**Authors:** Yang Hwan Kim, Hyeon Kim, Seong Cheol Park, Yongjin Kwon, Kyunghoon Yeom, Wosik Cho, Taeyong Kwon, Hyeok Yun, Jae Hee Sung, Seong Ku Lee, Tran Trung Luu, Chang Hee Nam, Kyung Taec Kim

**Affiliations:** 1 Center for Relativistic Laser Science, Institute for Basic Science, Gwangju, 61005 Republic of Korea; 2 Department of Physics and Photon Science, Gwangju Institute of Science and Technology, Gwangju, 61005 Republic of Korea; 3 Advanced Photonics Research Institute, Gwangju Institute of Science and Technology, Gwangju, 61005 Republic of Korea; 4 Department of Physics, The University of Hong Kong, SAR Hong Kong, China

**Keywords:** High-harmonic generation, Ultrafast photonics

Correction to: *Nature Communications* 10.1038/s41467-023-38087-3, published online 22 April 2023

The original version of the Supplementary Information associated with this article contained an error in Supplementary Fig. S17 in which panel b was cut. The HTML has been updated to include a corrected version of the Supplementary Information.

## Supplementary information

Updated Supplementary Information

