# Supplementary material for: High-harmonic generation from a flat liquid-sheet plasma mirror
Source: Nat Commun. 2023 Apr 22;14:2328. doi: 10.1038/s41467-023-38087-3 (PMC10122666; doi:10.1038/s41467-023-38087-3)
Supplement: Supplementary file 1 — Supplementary Information [file 41467_2023_38087_MOESM1_ESM.pdf]

Supplementary Information for

High-harmonic Generation from a Flat Liquid-sheet Plasma Mirror

Yang Hwan Kim[1], Hyeon Kim[1,2], Seong Cheol Park[1,2], Yongjin Kwon[1,2], Kyunghoon Yeom[1,2], Wosik Cho[1], Taeyong Kwon[1,2], Hyeok Yun[3], Jae Hee Sung[1,3], Seong Ku Lee[1,3], Tran Trung Luu[4], Chang Hee Nam[1,2], and Kyung Taec Kim[1,2*]

[1]Center for Relativistic Laser Science, Institute for Basic Science, Gwangju 61005, Republic of Korea

[2]Department of Physics and Photon Science, Gwangju Institute of Science and Technology, Gwangju 61005, Republic Korea

[3]Advanced Photonics Research Institute, Gwangju Institute of Science and Technology, Gwangju 61005, Republic of Korea

[4]Department of Physics, The University of Hong Kong, SAR Hong Kong, People's Republic of China

*kyungtaec@gist.ac.kr

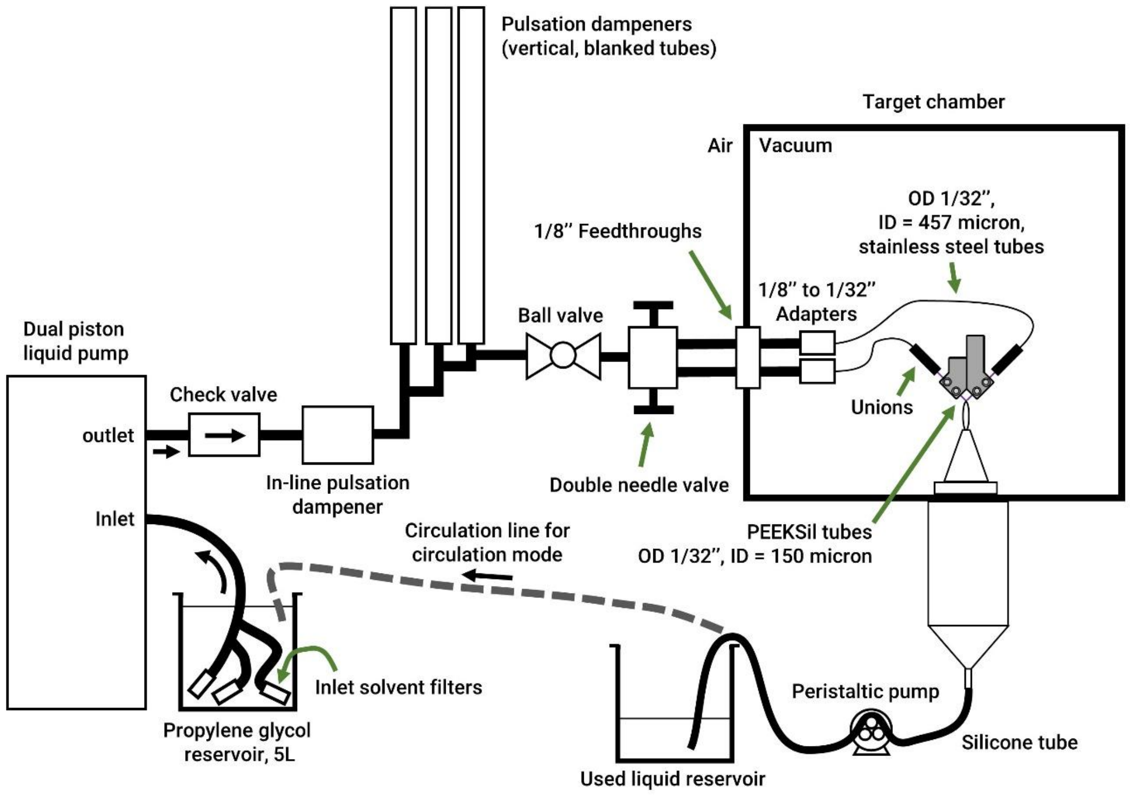

Fig. S1. Schematic drawing of liquid connection (not to scale). The degassed propylene glycol was stored in a stainless beaker with a volume of 5 L. The dual piston liquid pump fills up the propylene glycol through the inlet solvent filters with a pore size of 2 μm. In order to prevent backflow, the outlet of the liquid pump was connected to the check valve. The two kinds of pulsation dampeners reduced the fluctuation of liquid pressure and stabilized the flow of the liquid. The ball valve turns on and off the flow. The double needle valve was used to match the flow rate through the two capillaries. The collided liquid jets fell and collected in the catcher. The collected liquid in the catcher was pumped out to another reservoir in the air using peristaltic pumps. The used liquid was stored in another liquid reservoir with a volume of 5 L. For the longer operation of the liquid target, the used liquid can be circulated back to the fresh liquid reservoir (dashed line).

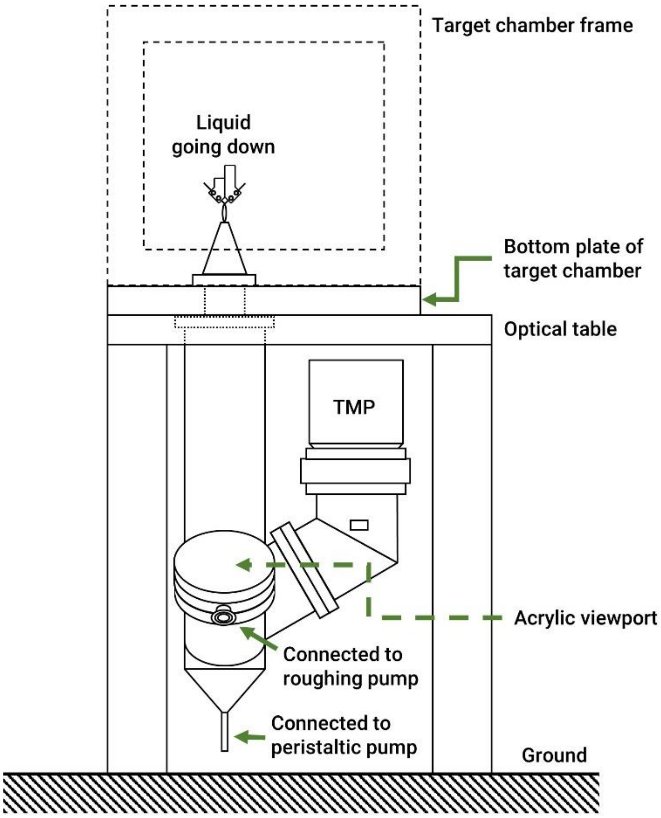

Fig. S2. **Schematic drawing of liquid catcher (not to scale).** The target chamber above the optical table was pumped out by a turbo molecular pump (Pfeiffer Vacuum GmbH, HiPace® 400). The roughing pump connected to the catcher was used as a roughing pump for the target chamber for better stability. A turbo molecular pump (Pfeiffer Vacuum GmbH, HiPace® 300) was attached to the catcher aside in order to avoid splatter of the liquid. The liquid was collected at the bottom of the catcher and pumped out by peristaltic pumps.

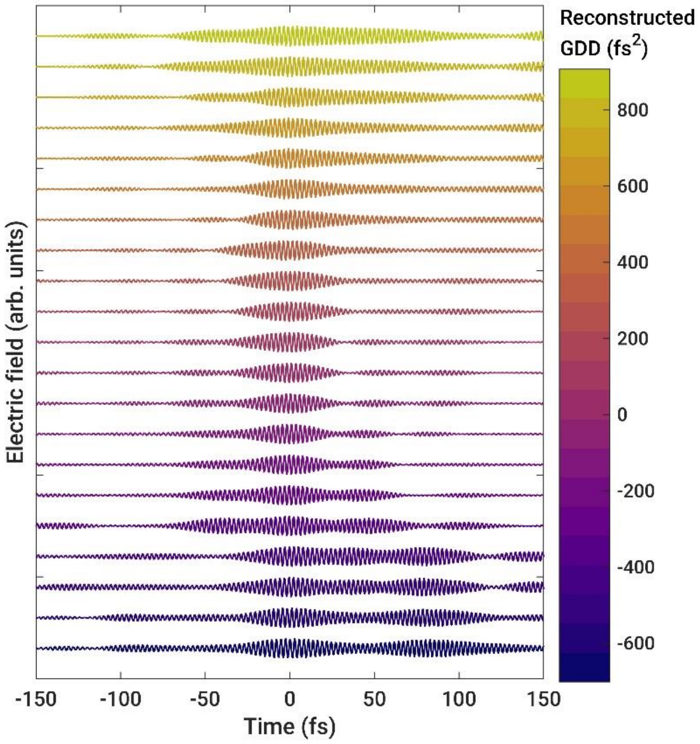

Fig. S3. Temporal profiles of laser fields measured using TIPTOE at different group delay dispersions (GDDs). For each line of plot, an offset was added for better visibility.

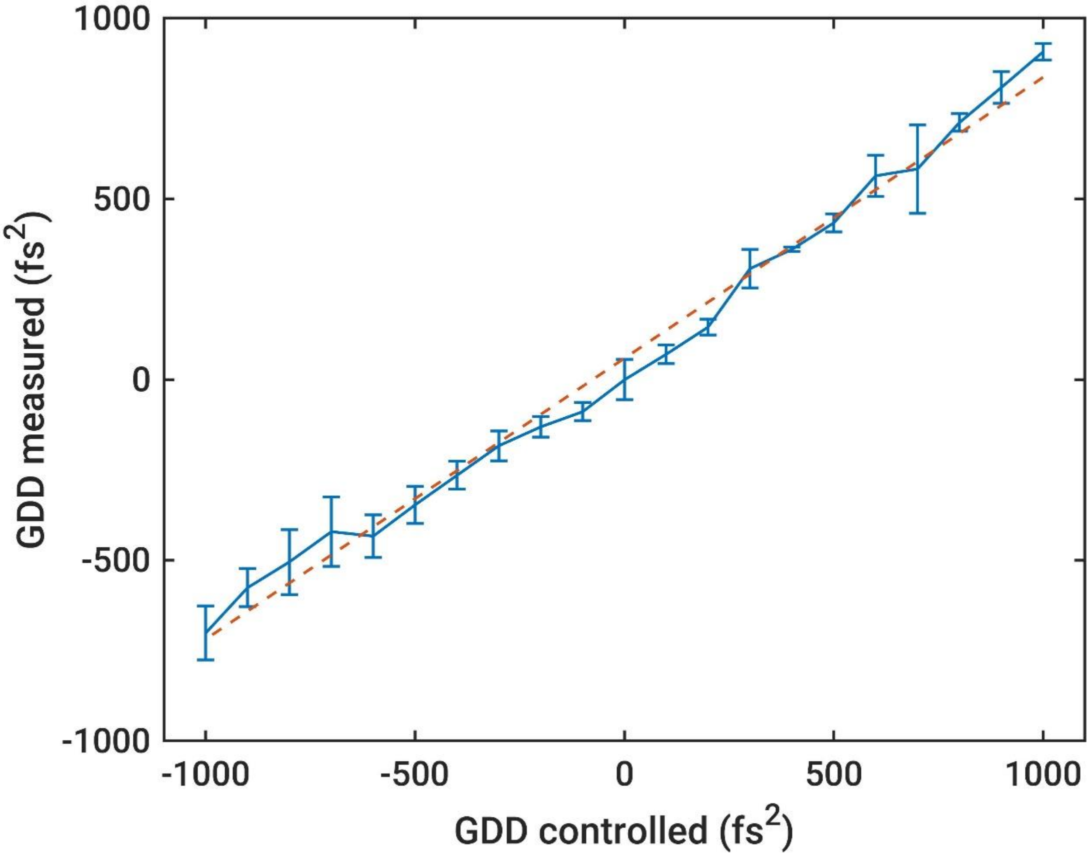

Fig. S4. GDDs obtained from the TIPTOE measurement. The error bars are the standard deviations of five independent measurements. The red dashed line is a linear fitting of data points.

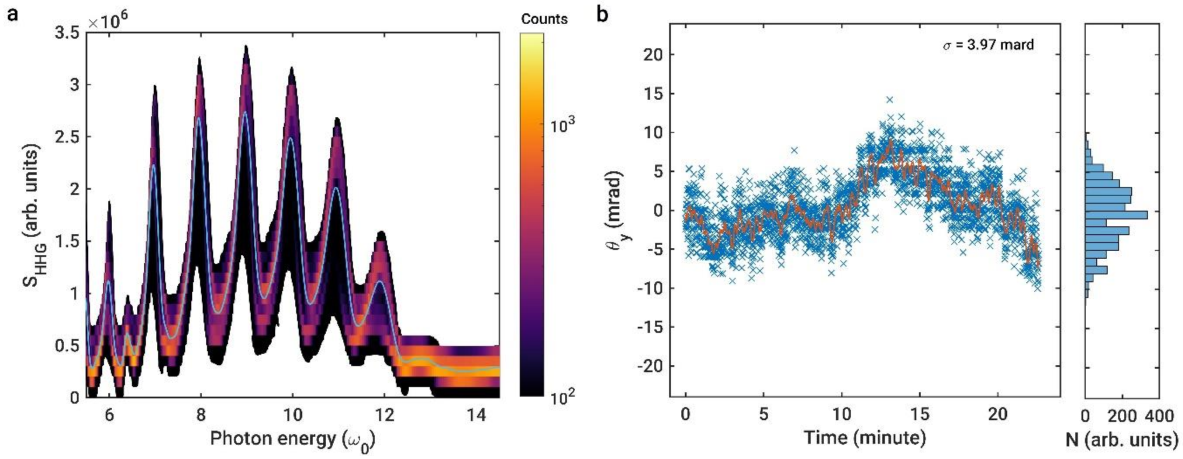

Fig. S5. Statistics of 1-kHz CWE harmonic over 22 minutes. a, CWE spectra (1357 spectra) taken with an exposure time of 101 ms. The color code shows the number of the spectrum overlapped. The time interval between each spectrum was around 400 ms to save the spectrum in a computer. **b,** Beam pointing statistics. The data points denote the position of the harmonic spectrum (θ_y) for the spectral range from $6.5\omega_0$ to $14.5\omega_0$. The solid red line shows smoothed data points.

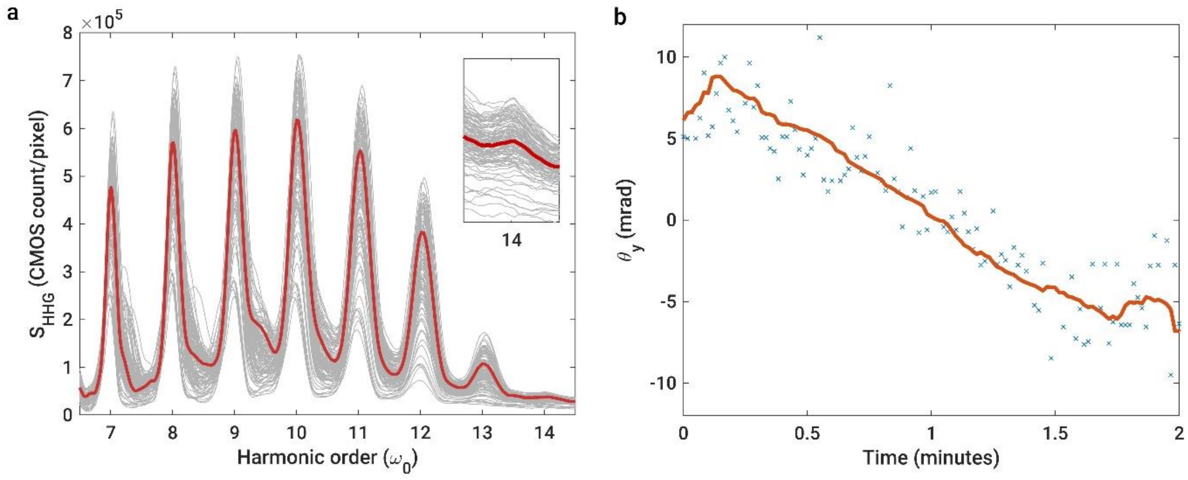

Fig. S6. Statistics of CWE harmonics obtained from a liquid plasma mirror recorded in a single-shot-based experiment. a, Spectral statistics of the single-shot CWE spectra obtained from a liquid plasma mirror. The solid gray lines are 120 single-shot spectra obtained in 2 minutes. The solid red line is the mean value of the 120 spectra. **b,** Beam pointing statistics of the CWE harmonic beam obtained from a liquid plasma mirror. Each data point corresponds to the angle where the maximum intensity is observed.

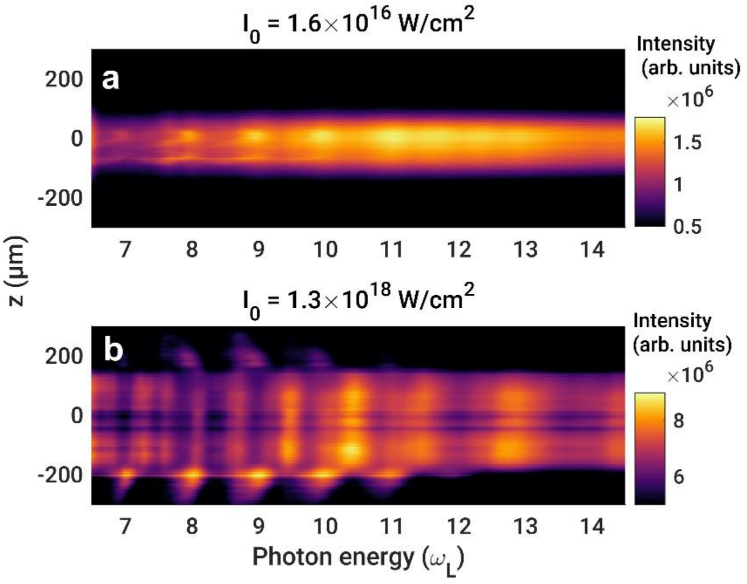

Fig. S7. Extreme ultraviolet spectrum from liquid plasma mirror using low temporal contrast laser pulses. a, b CWE spectrum recorded at each different target position z, parallel to the incident beam (see Fig. 1 in the main article) for the peak intensity of $I_0 = 1.6 \times 10^{16}$ W/cm^2 (**a**) and $I_0 = 1.3 \times 10^{18}$ W/cm^2 (**b**). The peak intensities were estimated at the foci, z = 0, where z = 0 was estimated by the position z where the CWE spectrum was the brightest for $I_0 = 1.6 \times 10^{16}$ W/cm^2. The target is close to the OAP for negative z. The CWE spectrum was not observed for $-200\ \mu\text{m} < z < 150\ \mu\text{m}$ at the high intensity because of the low temporal contrast. For this reason, we performed the CWE experiments at z where the CWE spectrum was observable and the brightest.

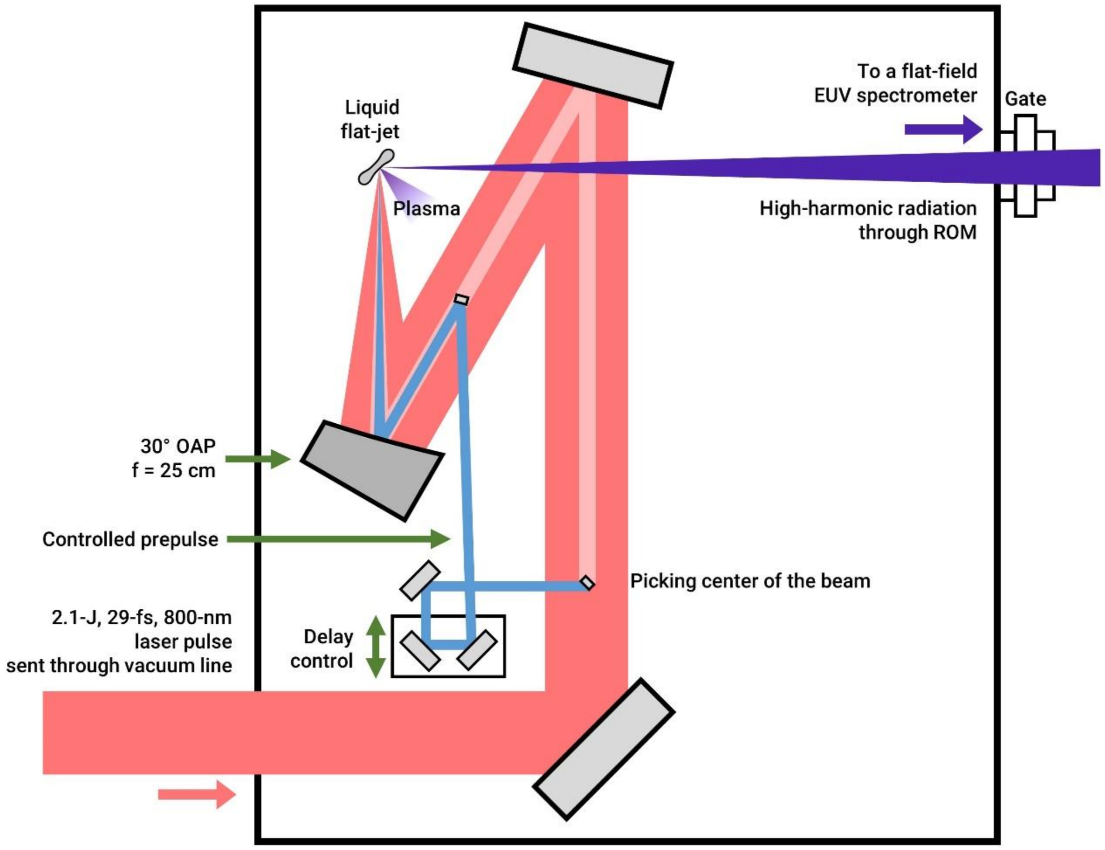

Fig S8. Schematic drawing of the beam path in the ROM experiments (not to scale). All the beam paths were in a vacuum. In order to make a controlled prepulse, a rectangular mirror was placed in the beam path. The delay between the main pulse and the prepulse was controlled by a motorized linear translation stage. The two beams were recombined by another rectangular mirror before an off-axis parabolic mirror (OAP). High-harmonic radiations were sent to a flat-field EUV spectrometer.

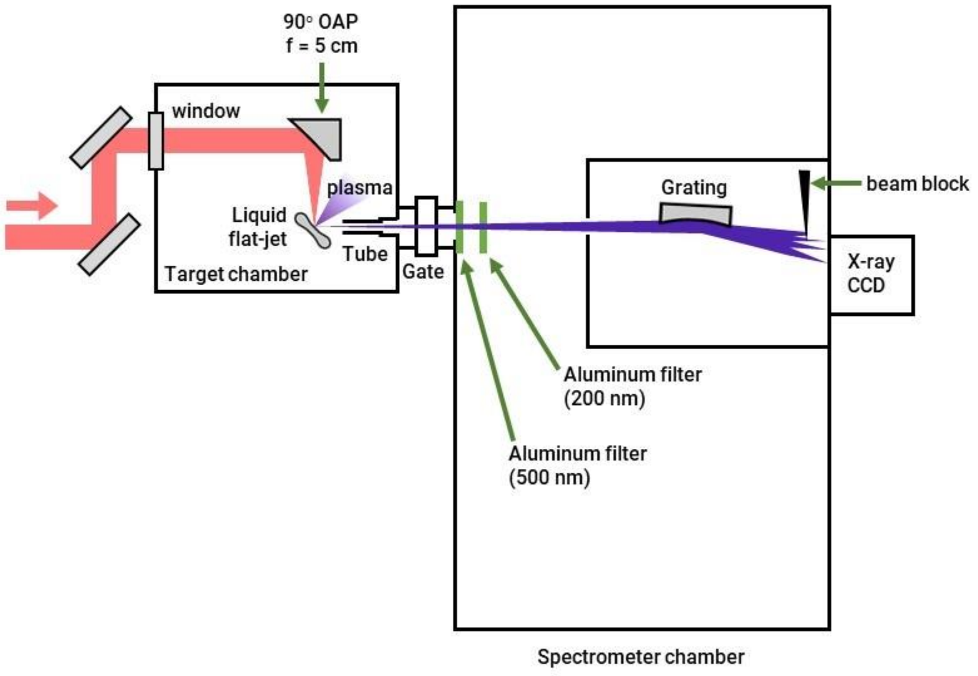

Fig. S9. Experimental setup for the photon number calibration using an X-ray CCD. To block the laser light completely, two aluminum filters were placed at the entrance of the spectrometer chamber. An aluminum blade was placed to block the specular reflection from the grating. The blade blocked some part of the diffracted beam, and the shadow was observed in the X-ray CCD. The counts recorded in the shadow were used to estimate the noise level of the measurement.

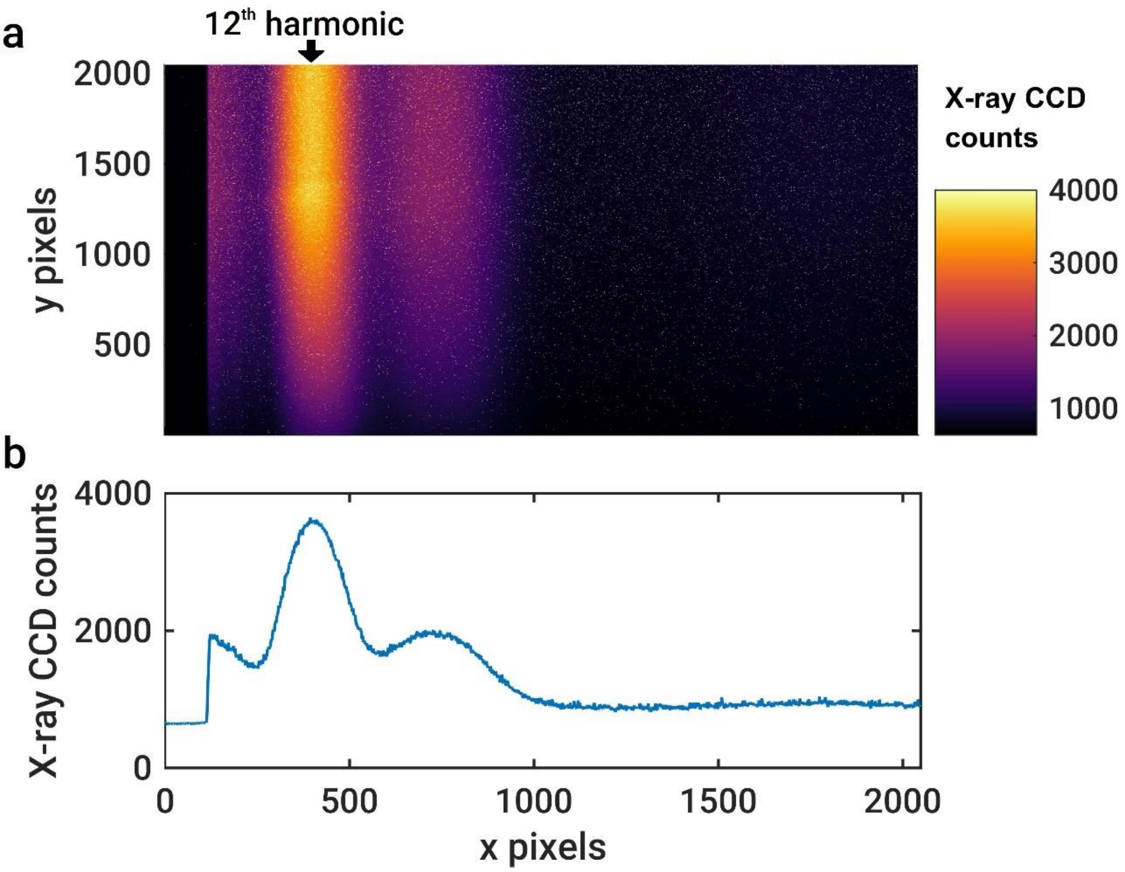

Fig. S10. Spectrum of CWE harmonics recorded using an X-ray CCD camera for photon number calibration. a, Raw image of an X-ray CCD camera. The 12th harmonics is denoted by the black arrow. Two Al filters were used, whose thicknesses are 200 nm and 500 nm. The black area recorded on the left side is the shadow of the beam block, which was placed after the EUV grating to block the specular reflection from the grating. The noise level of the CCD image was estimated by the counts in the shadowed area. **b**, Mean value of X-ray CCD counts.

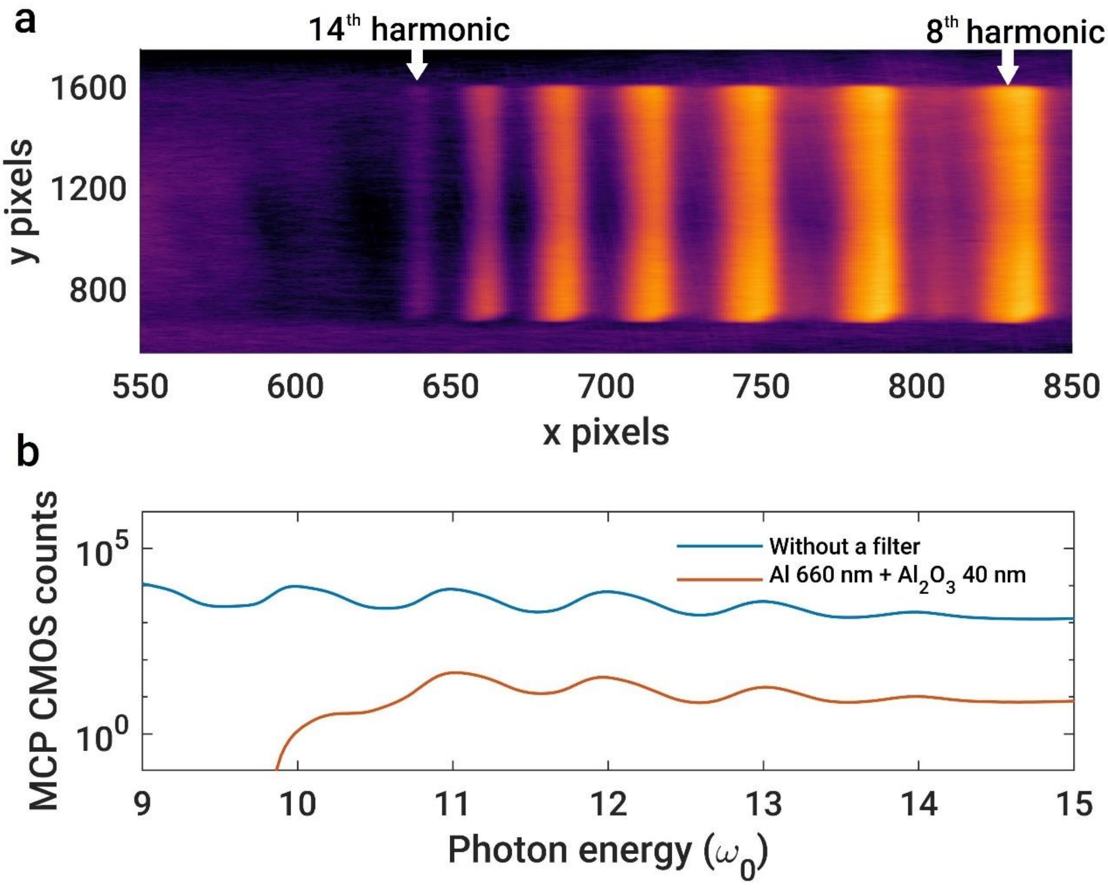

Fig. S11. Spectrum of CWE harmonics recorded by the MCP spectrometer for calibration. a, Raw image of the CEW harmonics obtained using the MCP spectrometer. The white arrows denote the 14th and the 8th harmonics. **b**, Spectrum obtained by the MCP spectrometer (solid blue line) and the spectrum filtered by Al filter with the thickness of 700 nm (solid red line). The total thickness of Al_2O_3 oxide layer to 40 nm was estimated for the two Al filters (i.e., 10 nm for each side). The transmittance data was taken from the CXRO database [B.L. Henke, E.M. Gullikson, and J.C. Davis. X-ray interactions: photoabsorption, scattering, transmission, and reflection at E=50-30000 eV, Z=1-92, Atomic Data and Nuclear Data Tables Vol. 54 (no.2), 181-342 (July 1993)]. The filtered spectrum was used to estimate the photon energy of the spectrum recorded by the spectrometer using the X-ray CCD camera.

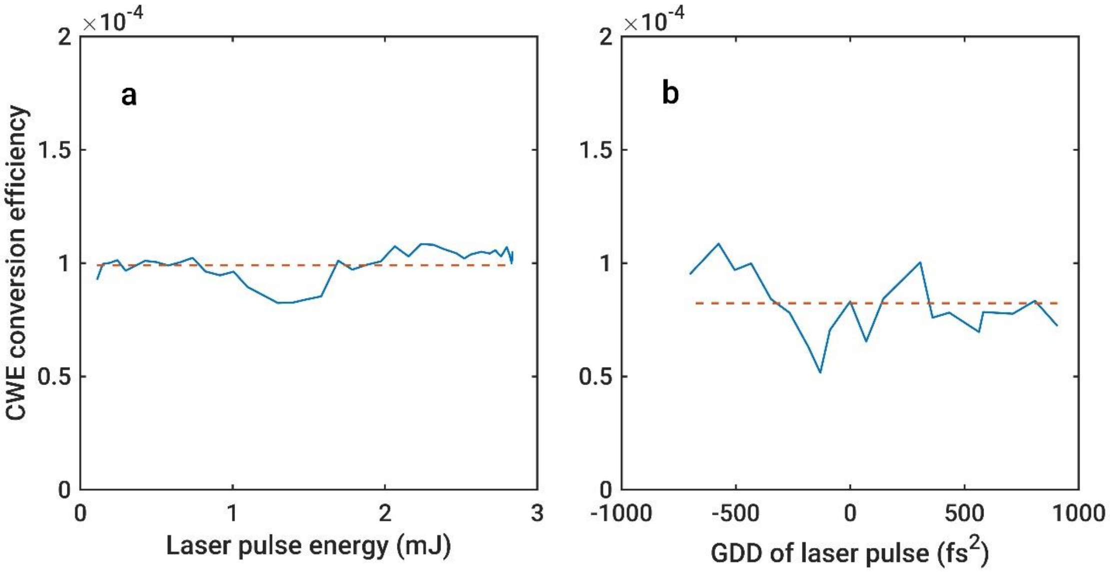

Fig. S12. Conversion efficiency of the CWE harmonics (7th–14th) from a liquid plasma mirror. a, Conversion efficiency of the CWE harmonics depending on laser pulse energies estimated from the experimental results shown in Fig. 4. **b**, Conversion efficiency depending on GDDs estimated from the experimental results shown in Fig. 3. The red dashed line show an average value.

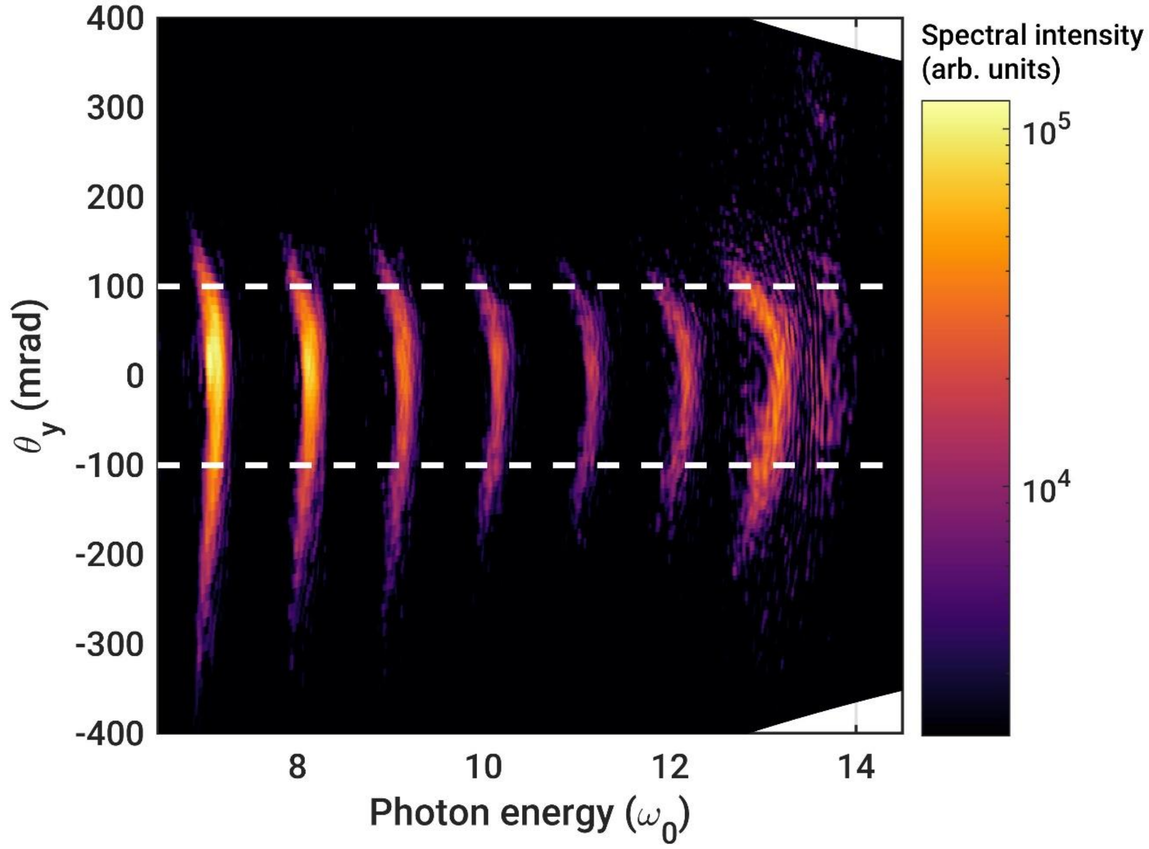

Fig. S13. Divergence of the CWE harmonic beam obtained using a 2D PIC simulation. The cone angle of the CWE harmonic beam was estimated to span ± 100 mrad (white dashed lines) from the result of a 2D PIC simulation. The size of the focal spot was 2 µm. The peak intensity of the laser pulse was 2×10^{17} W/cm^2 with a pulse duration of 30 fs. Within the cone angle of ± 100 mrad, 65% of the energy of the harmonics is contained in the PIC simulation result. This value was used to estimate the conversion efficiency of the CWE harmonics in the experiment.

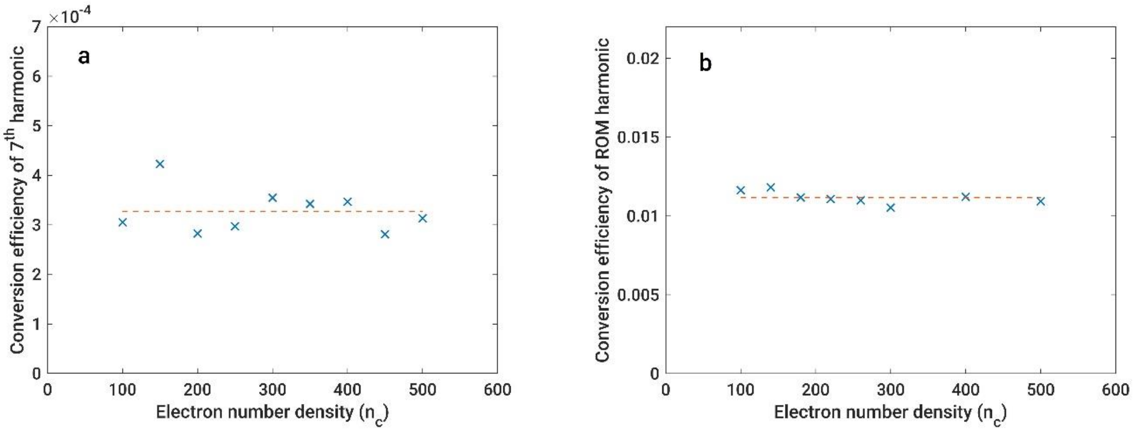

Fig. S14. Conversion efficiency of CWE and ROM harmonics estimated by PIC simulations for different electron plasma densities. a, Conversion efficiency of 7th order of the CWE harmonics for different electron number densities between $100n_c$ and $500n_c$, where n_c=1.7×10^{21} W/cm^2 is critical electron number density at the wavelength of 800 nm. The peak intensity of the driving laser pulse was 7.5×10^{16} W/cm^2, and the plasma scale length was $3\lambda/500$, where $\lambda = 800$ nm is the center wavelength of the laser pulse. **b**, Conversion efficiency of the ROM harmonics (10th–22th) for different electron number densities. The peak intensity of the driving laser pulse was 3.8×10^{19} W/cm^2. The plasma scale length was $\lambda/20$.

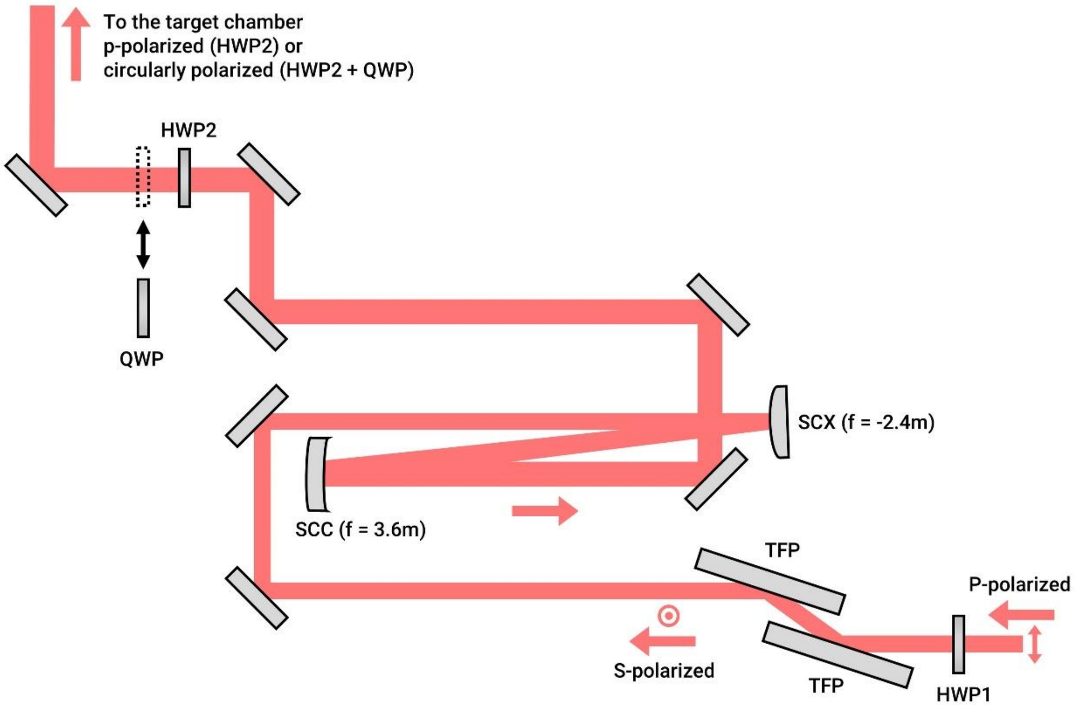

Fig. S15. Schematic drawing of beam path before the target chamber in the 1-kHz CWE experiments (not to scale). All the beam paths shown in this figure are in the air, not in a vacuum. HWP1: half-wave plate for energy control, HWP2: half-wave plate for polarization control, TFP: thin film polarizer, SCC: spherical concave mirror, SCX: spherical convex mirror, QWP: quarter-wave plate.

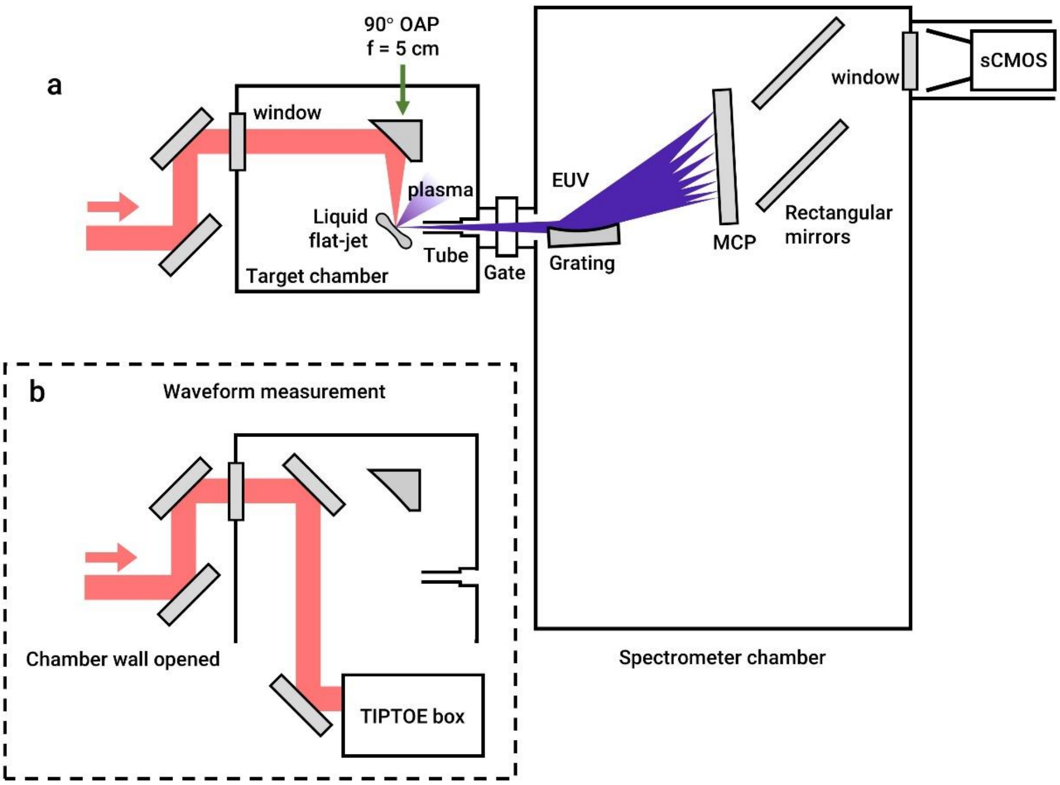

Fig. S16. Schematic drawing of the beam paths in the target chamber and the spectrometer chamber in the 1-kHz CWE experiments (not to scale). a, Beam path in the liquid target chamber and the EUV spectrometer chamber. Rectangular mirrors in the spectrometer chamber reflect the image of the phosphor screen of the MCP to the sCMOS camera. The tube is stainless steel with 1/4 inch of outer diameter for differential pumping between the target chamber and the spectrometer chamber. **b,** Beam path when the temporal waveform of the laser field was measured by the TIPTOE. The side wall of the chamber was open during the measurements. Waveforms for 21 different GDDs of the laser field were measured five times for each GDD value.

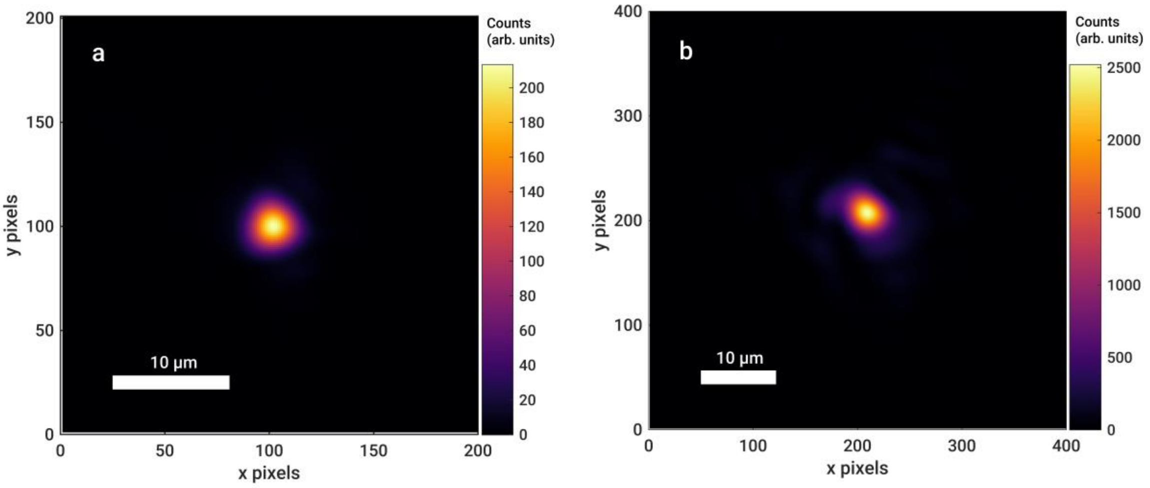

Fig. S17. Focal spot images. a, Focal spot image of the laser beam in the 1-kHz CWE experiment. The effective radius $\left(\int \sqrt{x^2+y^2} I(x,y)\mathrm{d}x\mathrm{d}y / \int I(x,y)\mathrm{d}x\mathrm{d}y\right)$ of the focal spot was estimated to be 2.8 µm. **b,** Focal spot image of the laser beam in the ROM experiment. The effective radius of the focal spot was estimated to be 6.0 µm.
